# From Universal Aspiration to Precision Immunization: A Hypothesis Framework for Antigen-Defined, HLA-Restricted Cancer Vaccines

**DOI:** 10.3390/vaccines14090753

**Published:** 2026-08-29

**Authors:** Sarfaraz K. Niazi

**Affiliations:** College of Pharmacy and Pharmaceutical Sciences, Washington State University, Spokane, WA 99202, USA; s.niazi@wsu.edu

**Keywords:** cancer vaccine, neoantigen, mRNA, shared tumor-specific antigen, immunopeptidomics, HLA, immunodominance, molecular residual disease, cancer interception

## Abstract

Cancer vaccination addresses four distinct problems: preventing oncogenic infection, intercepting premalignant clones, clearing molecular residual disease, and treating established cancer. This hypothesis framework proposes seven sequential gates for antigen-defined, HLA-restricted T-cell vaccines: tumor specificity, natural peptide–HLA presentation, population coverage, persistence under immune selection, selective T-cell recognition, manufacturability, and randomized clinical benefit. The proposed architecture combines a pre-manufactured core of validated shared antigens with a personalized shell when shared targets do not cover the tumor or the patient’s HLA type. Current evidence supports restraint. Individualized mRNA neoantigen therapy with pembrolizumab produced a recurrence-free-survival signal in a randomized phase 2b melanoma trial, whereas a 20-antigen shared cassette produced immunodominant responses toward encoded TP53 epitopes rather than the KRAS neoantigens carried by the tumors. No quantitative coverage or persistence score is presented because the required population frequencies and persistence measurements cannot be supplied reliably from the published record. Four falsifiable predictions define the experiments needed to test presentation-first selection, antigen persistence, escape-route independence, and biomarker-directed treatment. The framework is therefore a research agenda, not a validated decision tool.

## 1. Introduction: Reframing the Cancer-Vaccine Problem

The term ‘cancer vaccine’ encompasses various biologically distinct interventions that share only a common effector mechanism. Prophylactic vaccines, such as those for hepatitis B and human papillomavirus, serve to prevent infections that are causal factors in cancer development. Therapeutic vaccines aim to educate the immune system to recognize and respond to antigens already expressed by malignant cells. Interception vaccines target premalignant clones before invasive disease begins. In situ approaches involve converting an existing tumor into its own source of antigen. These interventions vary in their targets, acceptable risks, clinical endpoints, and evidentiary standards. Grouping them under a single category has led to both exaggerated claims and poorly matched clinical trials [1,2,3,4].

The fundamental biological principle is that no single cancer antigen exists. Malignant transformation occurs through mechanisms such as viral oncogenesis, point mutations, insertions and deletions, frameshifts, fusions, modified splicing, epigenetic derepression, and lineage programs. Within a single patient, tumor subclones may exhibit diverse antigenic profiles and varied capacities for HLA presentation. Consequently, an effective framework must be based on a patient–tumor–HLA context rather than on a specific tumor classification. For instance, pancreatic, colorectal, and lung cancers harboring the same *KRAS* mutation might share a target; however, two tumors originating within the same organ may lack any common beneficial antigen [5,6].

The essential immune cascade is well characterized. Competent antigen-presenting cells must acquire the tumor antigen, present it on class I and class II HLA molecules, prime polyfunctional CD8+ and CD4+ cells, generate immunological memory, traffic into the tumor microenvironment, and sustain activity despite checkpoint signaling and suppressive myeloid networks [3,7]. A sequence-anchored library perspective introduces a more rigorous criterion: a pre-manufactured product should enter inventory only if its antigen is recurrent within a designated population, shows no evidence of presentation on critical normal tissues, is naturally expressed on tumor cells, is sufficiently clonal, and resists tumor elimination [8]. Collectively, these perspectives elevate vaccine development from basic platform engineering to a meticulously regulated chain of biological validation.

This article sets out that chain explicitly. It separates the four objectives, states the criteria a candidate antigen must satisfy, derives a core-and-shell architecture from those criteria, classifies the available evidence along two independent axes, and states four predictions together with the experiments that would falsify them. It is a hypothesis article. Its central claims are untested, and Section 13 identifies its weakest point.

## 2. Scope, Approach, and Evidence Classification

This document constitutes a narrative synthesis and hypothesis article. It is not a systematic review; it lacks a registered search protocol, and it should not be interpreted as presenting a pooled estimate of effect. This distinction matters because the author selected the trials discussed in Section 9 based on the criteria below, rather than through duplicate screening aligned with a prespecified protocol.

We identified literature through PubMed and ClinicalTrials.gov, supplemented by the 2024 to 2026 proceedings of the American Society of Clinical Oncology, the American Association for Cancer Research, and the Society for Immunotherapy of Cancer, with the search closing in July 2026. Only peer-reviewed full publications are relied upon as evidence of effect; no conference abstract is used, and the trial registry is cited only for the status of an ongoing study. Reviews are cited for mechanistic context rather than as evidence of effect.

Where the argument advances a previously proposed sequence-anchored library by the current author, such dependence is explicitly declared at the point of use rather than being implied silently throughout the text [8].

Evidence is classified along two independent axes rather than on a single ordinal scale, because one scale cannot separate a positive randomized trial from a negative one. Table 1 sets out the axes, and Section 9 uses them. Axis 1 records the study design alone and is not a certainty rating. A phase 1 trial reporting safety and immunogenicity is a prospective nonrandomized study, and assigning its design category from its endpoints would conflate design with result. Axis 2 records the result domain rather than a rank, so a single study may carry several domain codes, and the direction of each result is recorded separately. This separation is meant to expose the error of promoting a peptide-recognition result into language that implies clinical benefit (Table 1).

The two-axis scheme organizes this article. It has not been externally validated and is not offered as a replacement for GRADE or any other grading system [9], which answers a different question: it rates certainty in an estimate of effect for a defined comparison and outcome, neither of which most of the studies discussed here supply, because their results are immunologic rather than comparative. Where certainty is at stake, Section 9 states the principal limitations of the randomized evidence; no formal Risk of Bias 2 or GRADE assessment was performed [10].

### 2.1. Relationship to Prior Work and to Existing Antigen-Discovery Platforms

Four components of this article were previously proposed by the author in earlier work and are not claimed as innovative: the pre-manufactured, HLA-matched library of shared tumor-specific sequences; the criteria for including a sequence in this library; the delineation of clinical settings based on disease state; and the fallback to personalized neoantigens when shared targets are insufficient [8]. Readers acquainted with that work are advised to interpret the target taxonomy in Section 4 and the library description in Section 8 as reiterations rather than novel propositions.

The following recent developments are noteworthy. The seven-gate antigen-to-benefit sequence turns the criteria into an ordered pass-or-fail protocol, with a clearly articulated response if a candidate fails at each gate, ensuring it is halted for a specific reason rather than downgraded based on subjective judgment. The two-axis classification renders a negative randomized trial interpretable as such. The authors explicitly articulate the hypothesis that core priming establishes an immunodominance hierarchy that subsequently degrades the personalized shell, along with the design of a four-arm trial intended to refute this architecture. The authors define independence of escape routes across five dependency layers, rather than assuming it solely based on gene identity. Section 6 delineates the persistence gate as a set of measurements, refrains from proposing any scoring system over these measurements, and reports that, at present, the necessary measurements cannot be retrieved from published reports for any examined antigen.

This framework complements and depends on the immunopeptidomics-integrated discovery platforms now in clinical use. NeoDisc combines matched tumor and germline exome sequencing, tumor RNA sequencing, and mass spectrometry immunopeptidomics in one clinical pipeline, prioritizing mutated, tumor-specific, viral, and noncanonical antigens, and annotates defects in antigen processing and presentation, including allele-specific HLA loss and loss of beta-2-microglobulin [11]. Platforms of this kind are the instruments by which Gates 1 and 2 are implemented. They establish what is presented on an individual patient’s tumor. They do not answer which sequences should be held as released inventory before a patient is identified, which is the question this article addresses.

### 2.2. What Is Established and What Is Proposed Here

A reader is entitled to understand, at each juncture, whether a statement constitutes a reported finding or a proposition introduced in this article. Table 2 delineates the two: the left column is supported by cited primary literature, and the right column comprises propositions of this framework, none of which have been empirically tested. These propositions are included because they are testable; Section 10 delineates what would serve to refute each of them. 

## 3. Four Distinct Vaccine Objectives

**Figure 1:**
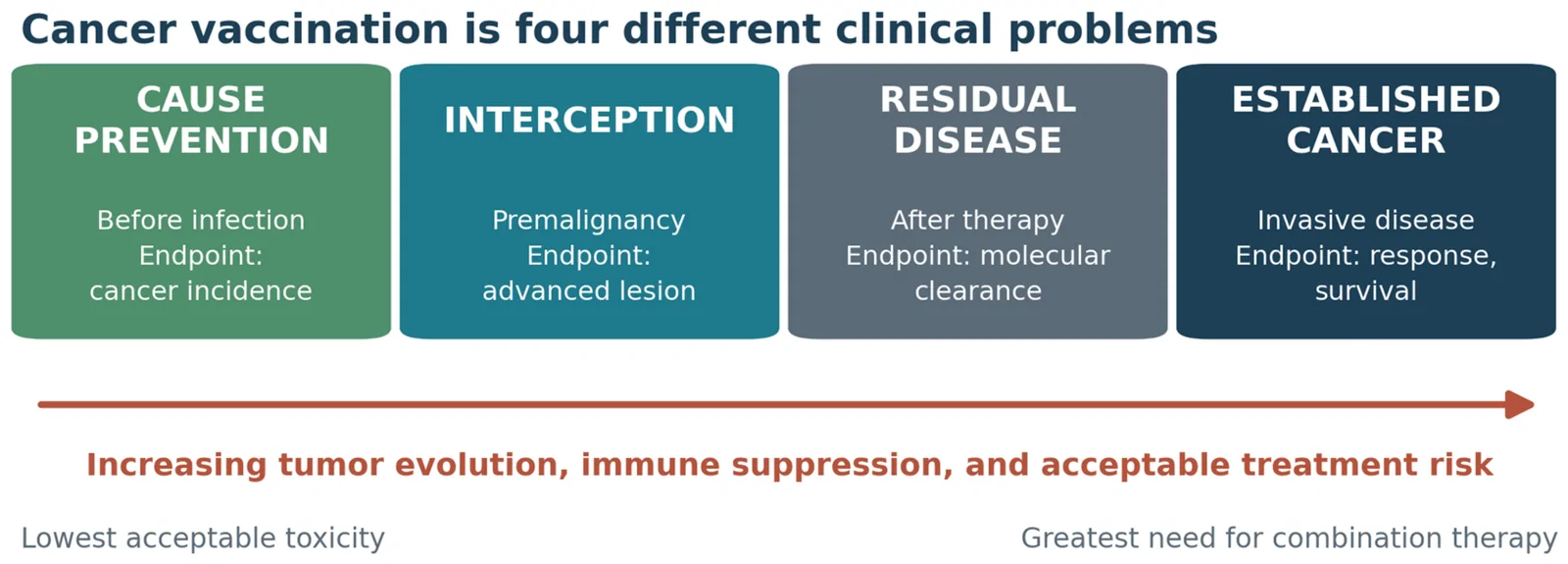
Four distinct objectives for cancer vaccines. The biological complexity, tumor progression, immune suppression, and acceptable treatment intensity escalate from the prevention of an oncogenic cause to the treatment of established cancer. Each scenario necessitates a different target, safety threshold, and clinical endpoint.

The four objectives do not represent points along a single continuum of difficulty; rather, they are distinct problems that share an effector mechanism. Biological difficulty, permissible treatment intensity, and the acceptable false-positive rate in antigen selection vary by disease state, and the endpoint must adapt accordingly. Cause prevention is treated as a boundary case and retained within the taxonomy to prevent conflation with therapeutic vaccination, which it explicitly aims to avoid. It is positioned outside the framework outlined in Section 5 for the reasons articulated in Section 3.1. (Table 3) (Figure 1).

### 3.1. Prevention of an Upstream Oncogenic Cause: A Boundary Case Outside the Gates

The most effective cancer-preventive vaccines act before malignant transformation by preventing a causative infection. The reduction in hepatocellular carcinoma following hepatitis B vaccination and the decline in invasive cervical cancer after human papillomavirus vaccination show that vaccination can prevent cancer, provided it targets a stable foreign causative antigen before immune escape occurs [1,2,12]. The model is comprehensive and specific, as the conditions necessary for its success, a foreign antigen, an intact immune system, and the absence of an established tumor microenvironment, are not present in the therapeutic context. The considerations in Section 5 question whether a peptide is presented by malignant cells on a patient’s HLA, the proportion of individuals capable of presenting it, and its ability to evade immune selection within a tumor, none of which are relevant prior to tumor development. From Section 4 onward, all discussions pertain to antigen-specific, HLA-restricted vaccines aimed at cells that are already malignant or pre-malignant.

### 3.2. Interception of Premalignancy

Interception occurs at the intersection of inherited risk and invasive cancer, where the shared-antigen hypothesis finds its strongest support. A germline *BRCA1* or *BRCA2* variant indicates increased risk but does not constitute a tumor antigen, as it is present in normal cells. Conversely, mismatch-repair deficiency produces frameshift peptides within premalignant lesions that recur across individuals, differ from the normal proteome, and evoke an immune response [20,21]. This target class is characterized by mechanism rather than convenience. The NOUS-209 program has translated this logic into human applications: an off-the-shelf vaccine using gorilla adenoviral and modified vaccinia Ankara vectors encoding 209 shared frameshift peptide neoantigens proved safe and elicited neoantigen-specific CD8+ and CD4+ responses in all 37 evaluable carriers of Lynch syndrome, who were maintained on retreatment [15]. The trial was conducted as a single-arm study; thus, the absence of advanced adenomas at the conclusion of the study colonoscopy should be regarded as an uncontrolled exploratory observation within a population under intensive monitoring. Tumor-directed immune recognition was demonstrated against engineered target cells, rather than naturally antigen-positive autologous tumors, and prophylactic application in individuals without cancer necessitates the highest safety standards in oncology.

### 3.3. Elimination of Molecular Residual Disease

Molecular residual disease following definitive therapy is arguably considered the most advantageous near-term therapeutic environment, although this remains a hypothesis rather than a definitive conclusion. Tumor burden is minimized, the suppressive architecture is least developed, priming efficiency is enhanced, and molecular techniques can monitor recurrence [4]. Personalized mRNA vaccination in pancreatic cancer, mutant-*KRAS* amphiphile vaccination in pancreatic and colorectal cancers, and personalized neoantigen vaccination in resected melanoma and renal cell carcinoma support this perspective [18,22,23,24,25]. Two cautions temper this assertion: the sensitivity and lead time of circulating tumor DNA vary according to tumor type and metastatic site, and the validity of surrogacy is established in some diseases but not others [26]. Only the melanoma program provides randomized evidence of benefit.

### 3.4. Treatment of Established Disease

Bulky metastatic disease presents the most challenging scenario and has yielded the most instructive failures. Factors such as antigen heterogeneity, defective interferon signaling, allele-specific HLA loss, beta-2-microglobulin disruption, T-cell exclusion, hypoxia, suppressive myeloid populations, and regulatory T cells can each impede a vaccine-induced response from achieving tumor control. Development should not presume that merely increasing immunogenicity will suffice to surmount a mature suppressive ecosystem. The Phase III outcomes for MAGE-A3, rindopepimut, PROSTVAC, and tecemotide, along with the failure of the HLA-A2-restricted OSE2101 vaccine to meet its primary endpoint in the intention-to-treat population, collectively demonstrate that a plausible antigen and a measurable immune response are not necessarily indicative of clinical success [27,28,29,30,31].

## 4. A Unified Taxonomy of Vaccine Targets

The fundamental distinction does not lie in personalized versus off-the-shelf approaches, nor in peptide versus mRNA strategies. Instead, it pertains to the relationship among the antigen, normal tissue, and tumor survival. Tumor-specific antigens originate from events absent from the normal genome or proteome. Conversely, tumor-associated antigens are self-proteins that are overexpressed, lineage-restricted, developmental, or ectopically expressed; central and peripheral tolerance limits the available repertoire, and on-target off-tumor toxicity constrains the therapeutic window. The repeated failure of tumor-associated antigen vaccines in phase 3 trials stems from neglecting this distinction [3,4,32].

In this article, an antigen is considered tumor-specific only when the peptide sequence presented on HLA has no detected counterpart in the normal proteome within the tested evidence base. Absence from reference databases does not definitively confirm its complete absence from essential tissues, as coverage of the normal tissue immunopeptidome remains incomplete and mass spectrometry is insensitive at low abundance levels. The operational standard involves no detected presentation in normal tissues within a declared evidence base, followed by cross-reactivity testing against the normal human proteome and primary human cells, with the residual uncertainty clearly stated. The delineation is established at the peptide–HLA level rather than the gene level because cryptic translation, alternative splicing, and proteasomal processing can produce a normal-tissue peptide from a gene that appears tumor-restricted based solely on transcript abundance.

Four subclasses within the tumor-specific class exhibit distinct behaviors. Viral oncoproteins are the most promising shared therapeutic targets because their foreign sequences and their frequent necessity in maintaining the malignant phenotype increase the fitness cost of antigen loss. Recurrent driver neoepitopes such as mutant *KRAS* and *IDH1* R132H are tumor-specific at the mutant residue and are costly to eliminate when the driver is truncal, although driver mutations do not necessarily guarantee presentation. Frameshift-derived neoantigens provide shared non-self C-termini in mismatch-repair-deficient tumors and in mutant *NPM1* and *CALR* neoplasms and are highly immunogenic due to their maximal dissimilarity to self [21,33,34]. Fusion junctions are sequence-unique and can be naturally presented; for instance, the DNAJB1-PRKACA product is observed in fibrolamellar hepatocellular carcinoma. However, researchers must empirically demonstrate the presentation of such fusion peptides in each case rather than assume it based solely on the fusion transcript [35]. Recurrence at the DNA level does not in itself create an antigen; the peptide must be present on the malignant cell within the relevant HLA context.

Personalized neoantigens remain essential when tumors possess limited validated shared targets, a frequent occurrence. Sequencing tumor and matched normal DNA, tumor RNA, and high-resolution HLA typing produces a patient-specific candidate set that broadens clonal diversity [18,36,37,38], but it requires several weeks for manufacturing, has variable success rates, and depends heavily on predictive models. The aim is to employ each method in contexts where its informational value is maximized (Table 4).

## 5. The Antigen-to-Benefit Gates

**Figure 2:**
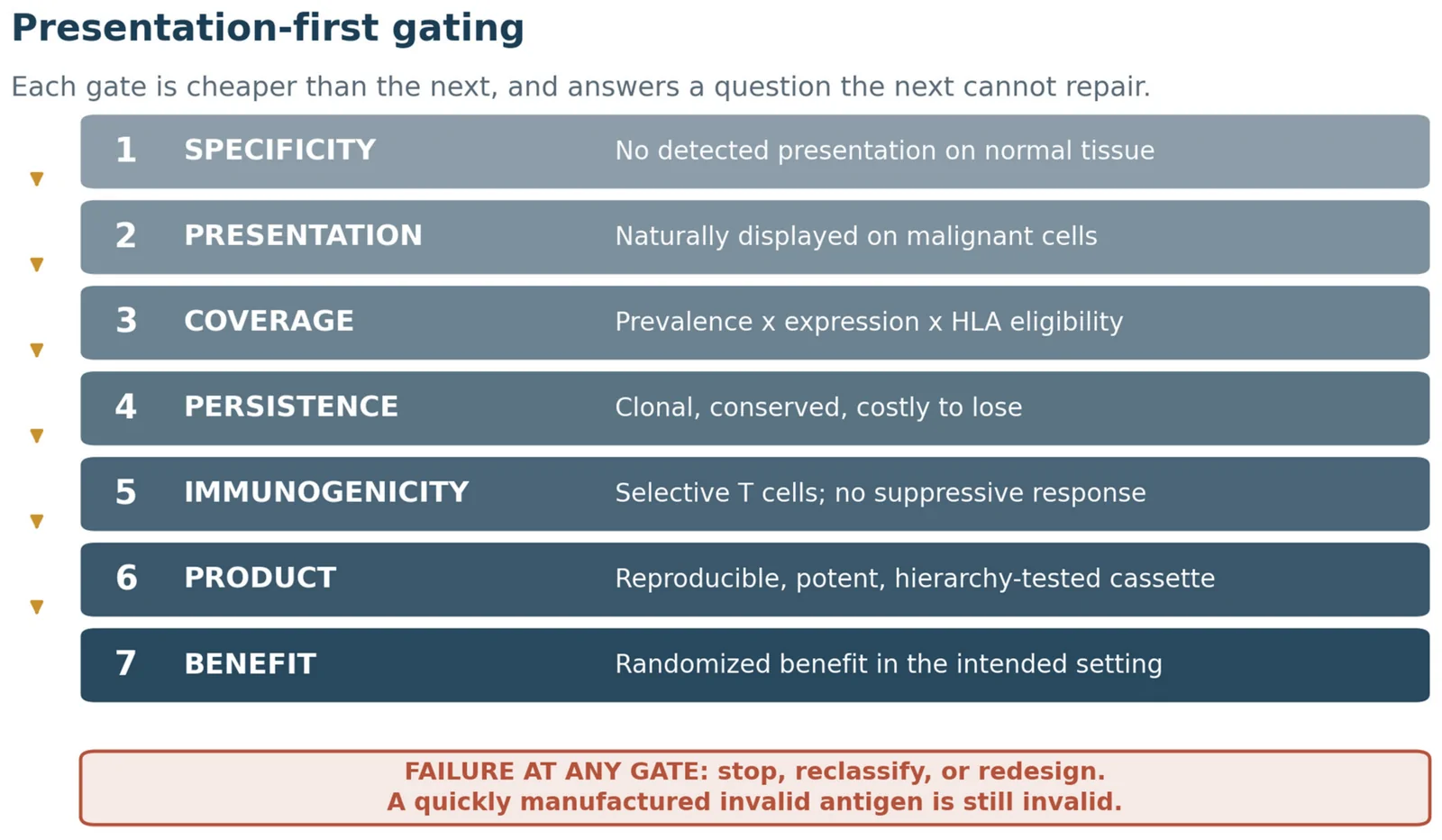
Presentation-first validation. Antigens progress solely through explicitly defined evidence checkpoints. Prediction serves as an initial step; the natural presentation and recognition of native tumor at endogenous antigen density constitute the bridging evidence. Gate 4 is delineated by the measurement specifications outlined in Section 6.

The gates below apply to antigen-defined, HLA-restricted vaccines targeted at malignant or premalignant cells and do not apply to prophylactic measures against oncogenic infections. A candidate must pass through seven sequential gates, and the sequence matters because each gate addresses a question the next cannot, and the cost of failure increases at every stage. The costliest error in developing cancer vaccines is including a biologically invalid antigen in the manufacturing process, because a swiftly produced invalid antigen remains an invalid product (Table 5, Figure 2).

### 5.1. Define the Intended Clinical State

A target product profile must delineate whether the product is intended for infection prevention, premalignant intervention, adjuvant treatment of molecular residual disease, or measurable cancer. It should specify the molecular subgroup, HLA eligibility, permissible manufacturing interval, standard-of-care backbone, anticipated combination partner, and clinically relevant endpoint. Absent this structured approach, identical immune-response outcomes may be misinterpreted as evidence of prevention, recurrence control, or tumor regression.

### 5.2. Obtain Representative Material and Discover Candidate Events

Personalized discovery necessitates the acquisition of paired tumor and normal DNA samples, tumor RNA, high-resolution HLA typing, pathological confirmation of tumor content, and meticulously controlled preanalytics. This process should include multiregional sampling or serial circulating tumor DNA analysis where heterogeneity is anticipated. For shared products, discovery cohorts must encompass multiple tumors, normal tissues, ancestries, HLA alleles, disease stages, and treatment histories, as a library derived from a limited ancestry or a small number of cell lines inherently reflects the biases of its source data, treating them as universal biology [39,40,41]. Candidate generation must incorporate substitutions, insertions and deletions (indels), frameshifts, fusion junctions, tumor-specific splice junctions, retained introns, viral proteins, and selectively post-translationally modified peptides. Orthogonal validation should be performed wherever a false positive could affect manufacturing processes, with mutant-allele expression demonstrated at the RNA level wherever tissue permits [42].

### 5.3. Move from Prediction to Natural Presentation

HLA-binding prediction functions as a filter rather than a validation step. Algorithms estimate processes such as cleavage, transport, binding affinity, complex stability, and occasionally immunogenicity; however, they cannot definitively establish that a peptide is generated within a tumor, reaches the appropriate loading pathway, and is presented densely enough for T-cell recognition. Monallelic immunopeptidome datasets and integrated models have enhanced predictive accuracy, although performance remains variable across different alleles and antigen classes [42,43,44]. Evidence of presentation should be evaluated and graded by its strength, yet Gate 2 remains a binary decision regarding advancement. A candidate is considered acceptable only if it is either directly detected in the primary tumor within the relevant HLA context or recognized through controlled conditions, demonstrating native, unmanipulated tumor recognition at endogenous antigen levels, with clear evidence of target specificity and HLA restriction. Detection in cell lines or engineered models serves only as supporting evidence, and failure to detect a low-abundance peptide should not be regarded as conclusive.

### 5.4. Rank by Specificity, Clonality, and Evolutionary Value

A valuable antigen is not detectably expressed in normal tissue, is expressed by a large proportion of malignant cells, is conserved across metastatic sites, and is tied to a function the tumor cannot easily discard, which is why truncal drivers and obligate viral oncoproteins are attractive. Subclonal passenger mutations may be immunogenic and still fail to control the tumor, because eliminating a minority clone can clear space for the majority. Clonality therefore needs an operational definition, and the simple one is inadequate. In a phylogenetic analysis of a single lung carcinoma with an *EGFR* mutation treated with tyrosine kinase inhibitors, radiotherapy, and a personalized neopeptide vaccine, the clonal *EGFR* exon 19 deletion was absent from a progressing, small-cell-transformed hepatic metastasis, and circulating tumor DNA showed that the wild-type clone predated vaccination and expanded during therapy [45]. That is a single case. The transferable lesson comes from the accompanying cohort analysis: mutations arising before whole-genome doubling are duplicated across copies and are substantially less likely to be lost at metastatic transition, so clonality should be defined relative to whole-genome doubling and copy number rather than by a high variant allele fraction in one biopsy, and persistent rather than baseline mutation burden is what tracks with sustained antitumor immunity [46].

Gate 3 necessitates a coverage calculation; however, this article does not perform such an analysis. Coverage is defined as the joint probability that a tumor possesses the event, expresses it, presents the peptide, and that the patient carries an HLA allele capable of presenting it. This measure should be reported for specific populations rather than as a global average because eligibility for HLA-restricted therapeutics varies by race and ethnicity, and class I allele frequencies differ significantly across populations [39,40,47]. Two essential requirements emerge. First, measure frequencies rather than assume them, using high-resolution allele and multilocus haplotype frequencies for specified population groups, available from donor registry data and reference resources that report population-specific frequencies [48,49]. Second, independence across the class I loci must not be presumed, since linkage disequilibrium among HLA-A, HLA-B, and HLA-C is considerable. Consequently, taking the product over per-locus terms will misstate the union of carrier events. An estimate of coverage derived from assumed frequencies under an independence assumption reflects the assumptions themselves and provides no definitive information about any specific population, which explains why none is reported herein.

### 5.5. Construct a Multivalent Set, and Test It for Dominance and Suppression

Multivalency should expand evolutionary coverage rather than merely increase the antigen count. The composition must encompass independent tumor clones, incorporate both class I and class II determinants, and avoid concentrating all targets within a single dispensable pathway. Increasing the number of antigens does not necessarily yield a superior product; the shared-library concept shows that encoded epitopes compete for immunodominance. In a Phase I trial involving a 20-antigen shared neoantigen cassette delivered via chimpanzee adenovirus prime and self-amplifying RNA boost, in conjunction with ipilimumab and nivolumab, T-cell responses were directed primarily at the encoded HLA-matched *TP53* epitopes. Conversely, responses against *KRAS* neoantigens in the patients’ tumors were diminished; no objective responses were observed, and the product was subsequently redesigned to focus solely on *KRAS* [14]. This trial underscores immunodominance and its association with a suppressed *KRAS*-targeted response; however, it cannot establish causation without a controlled comparison of cassette compositions. The fundamental design lesson remains consistent: composition influences which antigen elicits a response. Therefore, composition is a biological variable that should be tested within the relevant HLA background, rather than being regarded as a mere packaging choice.

Two additional mechanisms argue against maximal inclusiveness. Individual neoantigens may act as inhibitors rather than merely failing to induce immunity, and vaccine-induced neoantigen-specific Tr1 CD4+ cells can suppress the antitumor immunity the vaccine aims to establish [16,17]. Therefore, a multivalent construct must be evaluated for dominance hierarchy and suppression, with responses documented for each encoded target.

### 5.6. Choose the Platform After Choosing the Biology

The platform aligns with biological principles. Synthetic long peptides are chemically defined and demonstrate stability; however, they necessitate the use of an adjuvant and become impractical at high valency. DNA is stable and scalable but requires effective delivery mechanisms and nuclear entry. Dendritic-cell products enable controlled antigen loading, though at the expense of individualized production; for instance, sipuleucel-T has shown that cellular products can prolong survival, despite eliciting few objective responses in patients with metastatic castration-resistant prostate cancer [50]. Viral vectors induce strong priming effects but are constrained by anti-vector immunity, which heterologous prime-boost strategies can mitigate. Messenger RNA (mRNA) enables rapid, sequence-independent manufacturing, cytosolic antigen expression, and multivalency, with no intended mechanism of genomic integration, making it suitable for inventory libraries and personalized modules [51,52]. Its adjuvant property is a design parameter rather than an inherent feature; the degree and quality of innate stimulation depend on RNA chemistry, removal of double-stranded RNA impurities, formulation, and lipid composition [53,54]. The most effective construct is not necessarily the one that produces the highest amount of protein but rather the one that achieves the desired duration, localization, and quality of antigen presentation. Additionally, platform versatility cannot compensate for a biologically invalid antigen.

### 5.7. Link Potency, Combination, and Endpoint to Mechanism

Release testing encompasses the assessment of identity, sequence, integrity, purity, sterility, endotoxin levels, residual impurities, encapsulation efficiency, particle characteristics, and dosage, none of which establish biological functionality. A mechanism-linked potency assay must quantify at least one crucial step relevant to the product, such as the translation of the intended antigen, presentation of representative peptide–HLA complexes, activation of antigen-specific reporter cells, or stimulation of functional T cells. The immunodominance data emphasize that the cassette, rather than the module, should serve as the unit of biological comparability. Initiate combination therapy in response to diagnosed immune failure rather than following conventional approaches. Checkpoint blockade is justified when vaccine-induced T cells are inhibited by PD-1 signaling; however, it is not a universal solution. Tumors with allele-specific HLA loss, impaired interferon response, absent targets, physical exclusion of T cells, or dominant suppressive myeloid activity require alternative interventions, and PD-1 blockade in these contexts may increase toxicity without overcoming the underlying barrier [7].

Immune monitoring must be conducted in an orthogonal, prespecified manner and interpreted conservatively, as assays such as enzyme-linked immunospot, intracellular cytokine staining, peptide–HLA multimers, T-cell receptor sequencing, functional killing assays, and native tumor recognition each address different questions [23,36,55]. A critical inferential consideration requires explicit acknowledgment: in single-arm studies, an observed association between a robust vaccine-induced response and increased survival may merely indicate that healthier patients, with superior immune competence and lower disease burden, can generate such responses. Because immune competence itself is a prognostic factor, comparisons between responders and nonresponders do not establish mediation or causality. Endpoint selection should align with the disease context rather than the platform; for example, cancer incidence for preventive measures, advanced adenoma for interception within a controlled design, molecular clearance and recurrence-free survival for residual disease, and objective response and its duration for measurable disease. Circulating tumor DNA may serve as a valuable intermediate surrogate marker, with its validity established in some diseases but not universally [26]. Additionally, randomization must occur prior to interpreting any immune responses as definitive evidence of efficacy.

## 6. The Persistence Gate: What Must Be Measured, and Why No Score Is Proposed

One of the seven gates necessitates a decision procedure that the remaining framework does not provide. Gate 4 asks whether a target will endure immune and therapeutic selection, which is a measurement question rather than a ranking question, and it is addressed here with a measurement specification rather than a model. A candidate may exhibit strong immunogenicity and still fail therapeutically if it is subclonal, dispensable, or easily lost. This section proposes no score, index, or composite ranking. This section delineates which variables must be measured, the units of measurement, and the evidentiary standard against which they are assessed; Section 6.1 reports the outcomes when the published record is examined for these variables.

Persistence is an antigen characteristic within the malignant population, and it is distinct from the two preceding gates. Presentation evidence pertains to Gate 2, while cross-reactivity risk relates to Gate 1. Integrating either criterion into a persistence assessment would conflate the gates and undermine the sequential framework; therefore, both are enforced as pass-or-fail checkpoints based on the qualitative criteria outlined in Table 6 before evaluating persistence.

Four variables address the persistence inquiry. C represents the prevalence of malignant cells, adjusted for purity and ploidy, denoting the proportion of malignant cells exhibiting the event, with a credible interval provided. C is intentionally not termed clonality: a target expressed by fifty percent of the malignant population is not considered clonal, and a stringent clonality criterion is applied to the lower credible bound. M signifies the proportion of sampled metastatic or multiregion sites retaining the event, which requires the number of independent sites sampled for proper interpretation. D indicates functional dependence, determined via a specified external dependency measurement rather than inferred from driver status. R denotes presentation retention following a specified perturbation, expressed as the proportion of baseline presentation maintained; this terminology is employed deliberately, recognizing that processes such as complex half-life, antigen expression, proteasomal processing, HLA abundance, and the effects of interferon or the intended therapy are distinct and would be obscured if consolidated under a single term.

The four variables are not comparable. C and M represent proportions derived from sequencing, accompanied by reportable uncertainty; D is an inference based on functional-genomic evidence, with its strength varying depending on the target and lineage; and R is an experimental measurement that has no significance without the specific perturbation and assay used to generate it. Presenting these variables on a unified numerical scale would suggest an equivalence in their evidentiary validity that does not actually exist. Moreover, the second reason is particularly compelling: constructing a composite score requires exponents or weights, bin boundaries, and thresholds, all of which cannot currently be estimated from available data because no cohort exists in which the four variables have been measured against a durable control endpoint. Assigning values through subjective judgment results in a number whose order depends on the assignment process rather than on the inherent properties of the antigens. Furthermore, such several gains unwarranted authority once published, as it facilitates comparisons across different antigens, cancers, and programs, comparisons that the derivation process does not support.

The framework, therefore, terminates at the specification stage. Persistence constitutes a binary judgment, pass or fail, based on measured values compared against predefined thresholds, which are established in advance and justified within the context of the disease. Discrimination among candidates that meet the criteria is based on the measured values themselves, reported alongside their confidence intervals, rather than on a scalar score. This approach is less robust than a ranking score, but arguably more transparent. A gate defined solely by measurements can be demonstrated to be ineffective when relevant measurements are unavailable. Conversely, a score derived from assigned values can always be computed and will not indicate the absence of underlying evidence (Table 6).

One consequence of this specification challenges the library hypothesis proposed herein, and it is stated explicitly rather than implied. The shared frameshift class represents the group most susceptible to gate rigor. Its prevalence in mismatch-repair-deficient tumors is typically lower than that of an obligate viral oncoprotein or a truncal driver, because persistent mismatch repair failure results in indels throughout tumor progression rather than solely at its origin. Consequently, this class is the first to be excluded when a clonality requirement is applied to the lower credible bound. Evidence supporting its presentation often relies on engineered or reconstructed targets rather than on immunopeptidomic detection from primary tumors; thus, it is excluded a second time if Gate 2 is restricted to direct detection [15,21]. The gates, rather than any subsequent criteria, determine which antigen classes are preserved.

A prospective study, rather than further analysis of assigned values, would make a persistence composite legitimate. The four variables would need to be measured within a defined cohort, scored independently against a written specification, recorded as intervals, and incorporated into a time-to-molecular-recurrence model, in which any weighting is estimated from the data and compared with a model utilizing immunogenicity magnitude alone within the same cohort. A weighting derived from such a study would constitute an empirical result; any assertion made prior to this would not be.

### 6.1. Can the Persistence Variables Be Retrieved from the Published Record?

A specification is only valuable if the measurements it stipulates can be obtained. We preselected two traceable cases because of their exceptionally robust evidence base, reasoning that variables not retrievable in the most thoroughly documented cases are unlikely to be retrievable elsewhere. The first case involves the class I neoantigen *RPL18* p.Met53Ile, identified by mass spectrometry in the immunopeptidome of a primary cervical adenocarcinoma and shown to be immunogenic against autologous tumor-infiltrating lymphocytes from the same patient [11]. The second case pertains to mutant *KRAS* G12D presented on HLA-C*08:02 in pancreatic ductal adenocarcinoma [6,18,23]. A variable was deemed retrievable only when a published measurement, expressed in the units specified in Table 6, was identified, and no values were imputed (Table 7).

Both cases fail to meet the persistence requirement for the same reason. Presentation admissibility is satisfied for the first case and is arguable for the second. Functional dependence can be described qualitatively but not by the specified external dependency measurement required by the specification. Neither antigen reports malignant-cell prevalence as a cancer-cell fraction, adjusted for purity and ploidy, with a credible interval. Neither reports metastatic conservation across three or more independent sites. Neither reports presentation retention after a specified perturbation, the most relevant variable to the gate, in any form [11]. Two consequences arise, with the first being undesirable: Gate 4 cannot currently be applied to any antigen based on the published literature, and this is documented here as a negative result rather than incorporated into further analysis. The second consequence is more advantageous: the missing variables precisely define what measurements a prospective study would need to obtain, forming the core of Prediction 2. The search conducted was not systematic, and it is claimed that measurements in the necessary units were not identified, rather than asserting that none exist.

### 6.2. Status of the Persistence Specification

The specification is not a model; Section 6.1 emphasizes its more rigorous limitation: the variables it requires have not been reported in the requisite units for any examined antigen. It is provided because the gate it pertains to is currently addressed by a proxy that excludes the relevant variables, specifically binding affinity, which does not furnish information regarding malignant-cell prevalence or functional dependence. No evidence has shown that it surpasses this proxy in predictive capacity, nor does the proposal claim that it does. Instead, it explicitly states different variables, which constitutes the sole claim supported by the evidence.

## 7. Immune Escape and the Definition of Escape-Route Independence

The proposal of evolutionary trapping in Section 10 relies on the independence of escape routes; however, such independence cannot be presupposed because two antigens originate from distinct genes. Vaccine-induced immunity can be circumvented through at least seven mechanisms, which are not interchangeable.

Antigen loss eliminates the encoding event itself through mechanisms such as chromosomal instability, loss of heterozygosity, or the outgrowth of a pre-existing wild-type clone [45]. Allele-specific HLA loss results in the removal of the presenting molecule and consequently affects all epitopes restricted by that allele simultaneously. The loss of beta-2-microglobulin abolishes all class I antigen presentation. Defects in antigen processing eliminate epitopes that depend on the affected step. Defects in the interferon pathway reduce HLA inducibility and processing machinery, thereby diminishing effector function. T-cell exclusion obstructs effector access without altering antigen presentation. Additionally, suppressive differentiation, such as outcomes involving regulatory and Tr1 CD4+ T cells, transforms a vaccine-induced response into an inhibitory one [16].

Independence must therefore be established across five dependency layers, and a target pair counts as independent only when separable at each. Genomic independence requires that the two encoding events do not lie on the same segment vulnerable to a single deletion. Transcriptional independence requires that they do not share a regulatory mechanism vulnerable to a single epigenetic change. Processing independence requires that they do not depend on the same proteasomal or transporter components. Presentation independence requires restriction by different HLA alleles, and restriction within class I alone does not suffice, because loss of beta-2-microglobulin defeats both at once. Effector independence requires that the two responses are not suppressed by the same inhibitory mechanism. A class I driver epitope paired with a class II frameshift epitope is more independent than two class I epitopes on different alleles, and any test of evolutionary trapping must measure independence at each layer rather than presume it from gene identity.

### 7.1. HLA Class II Complexity: Help, Inertia, or Suppression

The emphasis on class I immunopeptidomics should not be interpreted as implying that CD4+ immunogenicity is uniformly advantageous. Class II neoantigens influence the magnitude, quality, and durability of the CD8+ response, and constructs that exclude them tend to produce weaker immunity [56]; however, CD4+ outcomes are variable: a vaccine-induced CD4+ response can be supportive, inert, regulatory, or actively suppressive, and neoantigen-specific cytotoxic Tr1 CD4+ cells hinder antitumor immunity rather than promote it [16]. Consequently, candidate ranking must differentiate between helper, cytotoxic, regulatory, and Tr1 responses rather than evaluating class II immunogenicity as a single positive measure. A class II epitope should not be accepted solely based on an interferon-gamma readout; it necessitates phenotyping of the induced CD4+ population and evidence that its induction enhances, rather than impairs, the CD8+ response within the same construct. A cassette that increases class II immunogenicity while transforming the CD8+ response into an exhausted or suppressed state has been detrimental, and an assay that reports only the response magnitude will erroneously record it as an improvement.

### 7.2. Antigen Density and the Limits of Engineered Targets

Recognition of a peptide does not equate to recognition of a tumor. The density of peptide–HLA complexes on primary tumor cells is frequently one to three orders of magnitude lower than that observed on peptide-pulsed targets, transduced high-expression lines, or monoallelic engineered systems, each of which addresses different research questions. Peptide-pulsed targets demonstrate that a T-cell receptor can recognize the complex, while engineered targets indicate that the epitope can be processed and presented when the antigen is abundantly expressed. However, neither model confirms that the epitope is presented at a sufficiently dense level on unmodified tumor cells to facilitate cytotoxicity. This distinction matters for the referenced work: the tumor-directed activity reported in the Lynch syndrome interception trial used engineered target cells expressing the frameshift peptide and HLA, rather than naturally antigen-positive autologous tumors [15]. The critical criterion is the effective destruction of unmanipulated, naturally antigen-positive primary tumors or organoids at endogenous antigen densities. Every antigen included in a screening library should meet this criterion, with the antigen density specified rather than inferred from an engineered surrogate.

## 8. The Core-and-Shell Vaccine Architecture

**Figure 3:**
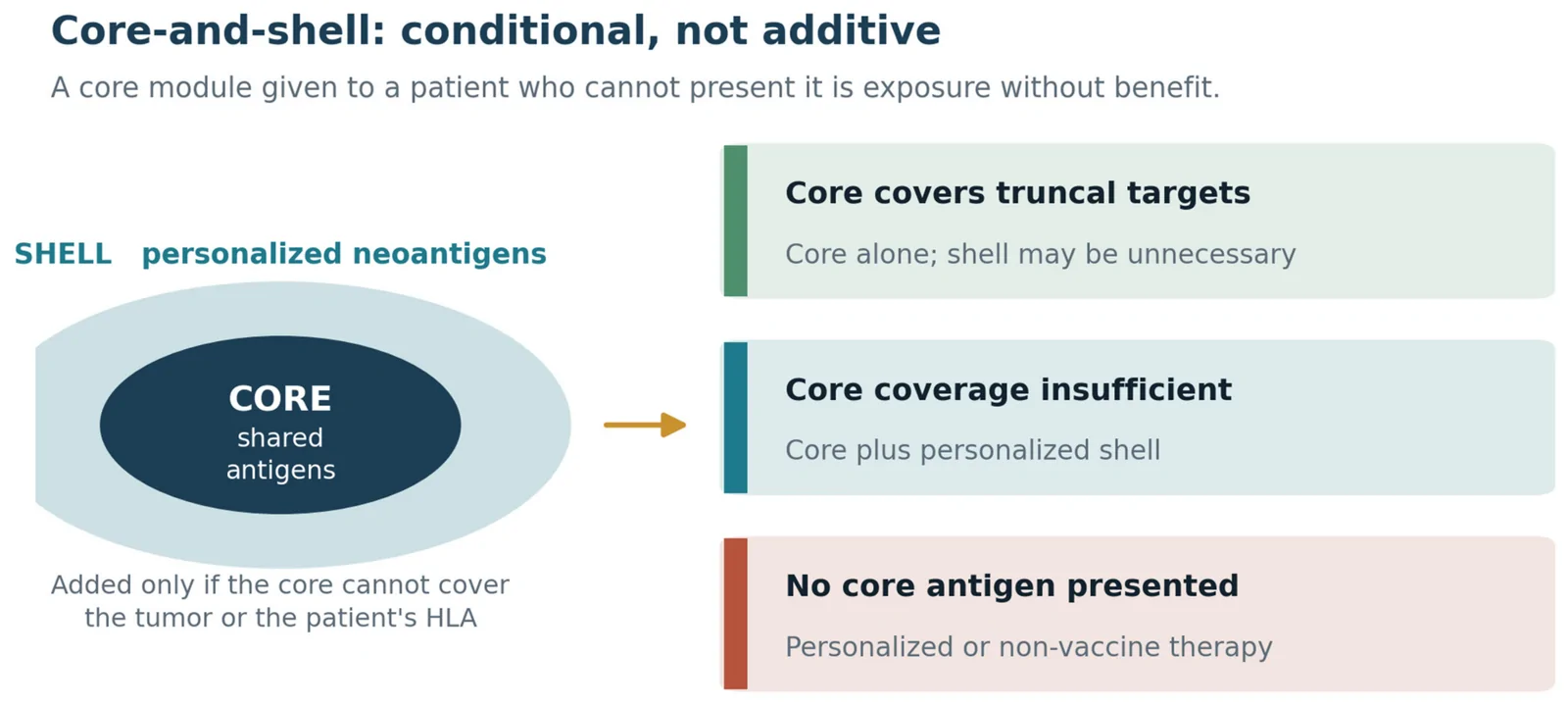
Core-and-shell precision immunization. A pre-manufactured, HLA-matched core covers validated, shared tumor-specific antigens, while a personalized shell is added only when private neoantigens are required. When no naturally shared target is present on the patient’s HLA, the patient proceeds to personalized or non-vaccine therapy. The sequencing depicted is a hypothesis; this section delineates the competing hypothesis under which core priming establishes an immunodominance hierarchy that diminishes the shell response.

The gates enumerated in Table 5 bear an architectural significance. Should shared antigens successfully clear gates 1 through 5 for a specified molecular subgroup, they may be produced proactively. Conversely, if such clearance is unattainable, personalization shifts from an elective option to an essential requirement. Consequently, a standardized system adopts a modular core-and-shell architecture (Figure 3).

The core comprises pre-engineered modules targeting recurrent, validated tumor-specific antigens. These modules are classified based on target event, HLA restriction, disease context, presentation evidence, and normal tissue assessment. It may also encompass selected viral oncoproteins, recurrent driver neoepitopes, shared frameshift products, altered C-termini, and validated fusion junctions. The shell consists of patient-specific neoantigens chosen from the individual tumor when the core does not offer sufficient clonal or HLA coverage [8].

The claimed operational benefit is separating inventory time from discovery time, and it must be stated precisely. A pre-manufactured core can remove the patient-specific manufacturing interval where a released module is available, and eligibility has been confirmed. It does not remove the time required for HLA typing, tumor-genotype confirmation, eligibility assessment, regulatory release, distribution, and scheduling. The advantage is the removal of one interval rather than the elimination of delay and reported manufacturing intervals indicate its size: a median of about twenty weeks from consent to vaccine availability in a phase 1 urothelial cancer trial [19], against several weeks in mRNA programs built on rapid synthesis [18,23].

A clinical precedent for this sequence exists and represents the most thoroughly documented approximation of an assessment of the architecture. In the TNBC-MERIT study, which explored individualized mRNA vaccination after standard therapy for early-stage triple-negative breast cancer, the first three patients received an off-the-shelf RNA lipoplex vaccine encoding shared, non-mutated tumor-associated antigens while their personalized preparations were being manufactured. Vaccine-induced, predominantly de novo T-cell responses were observed in nearly all fourteen evaluable patients, with eleven remaining relapse-free for up to six years [57]. Three limitations restrict the interpretability of these findings. First, the shared component consisted of non-mutated self-antigens rather than the validated tumor-specific antigens required by this framework’s core, making it incompatible with Gate 1 requirements. Second, the study employed a non-comparative design, with the bridging exposure involving only three patients. Third, the study reported no comparison of dominance between the shared and personalized components, which is crucial for evaluating the competing hypothesis outlined below.

The architecture is conditional, not additive by default. If the shared core already covers several truncal targets across both HLA classes, the shell may be unnecessary. If no core antigen is naturally presented on the patient’s HLA, the patient should proceed directly to personalized or non-vaccine strategies. Administering a core module to a patient who cannot present it is exposure without a plausible tumor-directed benefit, and eligibility rules should treat it as such.

The immunodominance finding presents a competing hypothesis that the underlying architecture must address, representing the proposal’s central risk. If epitopes within a cassette compete, epitopes across sequentially administered products may also compete. Early exposure to a shared core could establish an immunodominance hierarchy which subsequently compromises responses to the personalized shell, thereby rendering core followed by shell less effective than personalized vaccination alone, despite its earlier initiation. No existing trial addresses this concern. The necessary experiment involves a four-arm comparison: shell alone, core and shell combined, core then shell, and shell then core, with outcomes including antigen-specific clonal breadth, the dominance hierarchy across every encoded antigen, not solely responsive ones, native tumor recognition, and recurrence. Until such a trial is conducted, the sequencing hypothesis within this framework remains speculative and should not be asserted as fact.

A library ought to be managed as a governed evidence system rather than merely a repository of sequences. Each module should maintain version-controlled records of sequence data, molecular events, HLA restrictions, presentation evidence, and their respective coordinates on both axes of Table 1, along with data on normal-tissue evidence, immunogenicity, dominance behavior within its designated cassette, clinical exposure, manufacturing performance, and population-specific eligibility. It is important to explicitly state that, due to immunodominance operating at the cassette level, a core assembled from a different combination of modules does not constitute the identical product. Furthermore, a comparability protocol that considers module substitution as a minor modification is unlikely to be deemed valid considering immunodominance data.

## 9. Evidence as of July 2026

The clinical record supports a narrow conclusion: cancer vaccination demonstrates efficacy in specific contexts; however, it does not substantiate a universal platform or a pre-manufactured shared library. Personalized mRNA neoantigen therapy combined with pembrolizumab possesses the most robust randomized evidence pertinent to this domain. In the KEYNOTE-942 study, 157 patients with resected melanoma were randomized in a 2:1 ratio. At the prespecified primary analysis, the hazard ratio for recurrence or death was 0.561 (95% CI, 0.309 to 1.017), and the hazard ratio for distant-metastasis-free survival was 0.347 (95% CI, 0.145 to 0.828) [13]. Subsequent analyses at 34.9 and 60.3 months were descriptive and associated with nominal *p*-values; the later recurrence-free survival hazard ratio was 0.510 (95% CI, 0.294 to 0.887) [58,59,60]. Overall survival remains exploratory, and the confirmatory phase III trial is currently underway.

The broader randomized record presents mixed outcomes. Sipuleucel-T has demonstrated an improvement in overall survival among patients with metastatic castration-resistant prostate cancer; however, it is characterized as an autologous cellular product rather than an antigen-defined vaccine [50]. Five programs based on antigens or vectors, specifically tecemotide in START, MAGE-A3 in MAGRIT, rindopepimut in ACT IV, PROSTVAC in PROSPECT, and OSE2101 in ATALANTE-1 [27,28,29,30,31], failed to achieve their primary clinical endpoints. These unsuccessful trials suggest that immunogenicity, antigen expression, or HLA matching alone is insufficient to establish clinical benefit. This narrative synthesis was not conducted as a systematic review or subjected to a formal Risk of Bias 2 assessment [10].

Shared products demonstrate more substantial early evidence in the context of interception than in treatment, though exclusively regarding safety and immune recognition. NOUS-209, a fixed cassette encoding 209 shared frameshift peptides, elicited neoantigen-specific CD8+ and CD4+ responses in all 37 evaluable Lynch syndrome carriers; however, controlled clinical benefit and native-tumor recognition at endogenous antigen density remain unconfirmed [15]. ELI-002 induced mutant-KRAS-specific responses and biomarker reductions in a single-arm residual-disease study, yet requires confirmation through randomized trials [22,25].

The most informative shared-library result is a failure. In the phase 1 portion of SLATE, a 20-antigen cassette produced responses dominated by encoded HLA-matched TP53 epitopes, attenuated responses against the KRAS neoantigens carried by the tumors, and no objective responses; researchers subsequently redesigned the product around KRAS [14]. This result does not prove that TP53 responses caused KRAS suppression, but it establishes cassette composition as a biological variable.

Individualized programs have elicited immune responses or sustained immune-cell persistence in various cancers, including pancreatic, melanoma, renal, hepatocellular, urothelial, and triple-negative breast cancers, across multiple platforms such as mRNA, peptide, and DNA [18,19,23,24,57,61,62]. Autogene cevumeran has induced neoantigen-specific T cells in eight out of sixteen patients with resected pancreatic cancer, while ELI-002 has generated mutant-KRAS-specific responses in twenty-one of twenty-five patients [18,22,23,25]. These uncontrolled associations do not confirm clinical benefit. Therefore, the robust evidence remains clear: personalized vaccination has the strongest therapeutic evidence; shared vaccination shows promising immunogenicity and a single example of cassette-level failure; and, to date, no pre-manufactured shared mRNA antigen library has demonstrated clinical activity or advantage.

## 10. Four Testable Predictions

The core-and-shell architecture serves as the foundational framework. The four assertions outlined below constitute its testable predictions. Each assertion is articulated in a manner that permits refutation, and each is associated with an experiment designed to potentially disprove it.

### 10.1. Prediction 1: Presentation-First Development Will Outperform Prediction-First Development

The traditional methodology commences with recurrent mutations and utilizes algorithms to select epitopes. Conversely, the presentation-first development reverses this sequence: datasets of tumor immunopeptidomes across various HLA backgrounds are employed to identify recurrent peptide–HLA complexes, which are subsequently analyzed through genomic and transcriptomic data to determine their origin, clonality, and prevalence within populations. Presentation-first libraries are anticipated to contain fewer modules, with a higher proportion capable of inducing T cells that effectively eradicate native tumors at endogenous antigen densities. The definitive experiment involves a blinded comparison between equally sized presentation-first and prediction-first panels within matched HLA backgrounds, evaluated based on their ability to eliminate native tumors rather than peptide reactivity. Prediction is deemed unsuccessful if prediction-first panels attain equivalent recognition of native tumors per encoded target, indicating that existing algorithms have bridged the gap and that immunopeptidomics functions as a confirmatory tool rather than a selective one [42,43,44].

### 10.2. Prediction 2: Measured Antigen Persistence Will Predict Durable Control Better than Immunogenicity Alone

A candidate may exhibit strong immunogenicity yet fail therapeutically if it is subclonal or easily lost. The hypothesis posits that, among candidates that have already satisfied the criteria for specificity and presentation, the four persistence variables outlined in Section 6, measured rather than assigned, will demonstrate a stronger association with durable molecular clearance than binding affinity or the magnitude of enzyme-linked immunospot responses. It pertains to the variables themselves, not any specific method of combining them, and remains untested rather than supported, as Section 6.1 reports that these variables have not been measured in the necessary units. The hypothesis is invalidated if the response magnitude alone predicts durable control as effectively as the persistence variables in prospective testing with measured inputs; current data suggest that this outcome is less probable without entirely excluding it, since persistent mutation burden rather than baseline mutation load is linked to sustained antitumor immunity [46]. The critical evaluation involves incorporating these variables into a time-to-molecular-recurrence model, with weights estimated from the data, and comparing its concordance with a model utilizing immunogenicity magnitude alone within the same cohort.

### 10.3. Prediction 3: Evolutionary Trapping Requires Measured Escape-Route Independence

A vaccine may demonstrate increased durability when it targets two antigens whose simultaneous absence entails a significant biological cost. It is predicted that target pairs identified as independent across the five dependency layers discussed in Section 7 will impede clonal outgrowth more effectively than pairs matched for immunogenicity but exhibiting biological redundancy. Furthermore, the advantage is expected to correlate with measured independence rather than overall immunogenicity. A pair is considered not independent if the two antigens originate from different genes or bind different HLA alleles; for instance, two class I epitopes located on different alleles are lost simultaneously when beta-2-microglobulin is disrupted. Therefore, the most effective pairs combine class I with class II restriction or pair an HLA-dependent target with a non-HLA-dependent effector mechanism. The definitive experiment uses barcoded heterogeneous organoids and immune-competent models in which each dependency layer is individually perturbed. The hypothesis fails if redundant pairs similarly delay escape, indicating that the driving force behind outgrowth is immunogenic pressure rather than the topology of escape routes.

### 10.4. Prediction 4: State-Based Dosing and Diagnosed-Failure Combinations Will Outperform Fixed Regimens

Conventional schedules allocate a predetermined peak period and predefined augmentation points on a calendar. A state-based schedule monitors circulating tumor DNA, mutation-specific molecular residual disease, antigen-specific T-cell frequency, T-cell phenotypes, including regulatory and Tr1 markers, and antigen persistence, triggering interventions when molecular signals are detected or when functional immunity diminishes. The same rationale applies to combination therapies: without priming, innate or antigen-presenting cell support is needed; primed but exhausted T cells require checkpoint blockade; T-cell exclusion warrants stromal or vascular interventions; and loss of antigen presentation indicates the need to change targets. State-based assignment is predicted to improve the ratio of durable immune control to cumulative toxicity compared with fixed schedules and fixed combinations. The critical experiment compares a biomarker-guided arm with a fixed-schedule arm, with decision rules pre-specified in the protocol. Sensitivity and lead times of circulating tumor DNA vary across tumor types and sites; therefore, this hypothesis must be validated separately for diseases with analytically validated assays. The approach may fail if adaptive assignment does not improve the control-to-toxicity ratio, suggesting that the measured immune states do not correspond to actionable bottlenecks (Table 8).

## 11. Scientific Integrity and Falsifiability

Development must ensure distinctness concerning sequence accuracy, predicted HLA binding, natural presentation, vaccine-induced peptide reactivity, native-tumor recognition, molecular response, recurrence control, and survival. These factors are arranged along the outcome axis of Table 1 and are not interchangeable. A construct with correct sequencing may lack evidence of presentation; a naturally presented peptide may not elicit a beneficial response; and a strong response may not improve outcomes or could potentially suppress the intended response [16,17].

Antigen-selection algorithms, data sources, software versions, thresholds, HLA calls, manual overrides, and manufacturing attrition should be recorded prospectively, and every encoded target should be reported rather than only the immunogenic successes, because the denominator is where the design lesson resides: the immunodominance hierarchy in the SLATE cassette was discernible only because responses to all encoded antigens were measured and reported [14]. Immunologic assays require predefined positivity criteria, baseline samples, wild-type and irrelevant peptide controls, and orthogonal confirmation. Additionally, cross-reactivity should be evaluated against the normal human proteome and primary human cells, particularly in cases where the recipient does not have cancer.

Early trials should be characterized by their capacity to establish foundational insights. Phase 1 studies primarily assess feasibility, dosing, safety, and immunogenicity, and they rarely demonstrate a survival benefit. Moreover, comparisons between responders and non-responders are often confounded by differences in immune competence, which itself is a prognostic factor. Assertions of therapeutic benefit must be supported by rigorous methodology, including randomization, intention-to-treat analysis, appropriate control groups, predefined endpoints, and sufficient follow-up periods.

The four predictions should be revised or abandoned if they prove to be incorrect, and Table 8 delineates the conditions under which each prediction fails. Additionally, it presents the competing hypotheses that would undermine the validity of the entire framework. Falsifiability is not a flaw of a framework; rather, it is the attribute that renders it functional and useful.

## 12. A Practical Development Blueprint

A credible program commences with a singular molecularly defined use case rather than a broad pan-cancer assertion. It selects a specific disease state characterized by measurable residual disease or a clearly defined adjuvant endpoint, compiles representative tumor and normal datasets across diverse populations, identifies potential tumor-specific genetic events, confirms natural HLA presentation, and characterizes candidates based on the persistence variables outlined in Section 6 through direct measurement instead of pre-established thresholds. It then constructs a multivalent Class I and Class II cassette, which it evaluates for immunodominance hierarchy and suppressive outcomes within relevant HLA backgrounds. Platform selection and delivery methods are determined next, with potency correlated to the underlying mechanism and the choice of combination partner aligned with the tumor’s measured immune barrier. The eradication of native, unmanipulated tumor at endogenous antigen density, rather than peptide reactivity, precedes any randomized clinical trial.

Each module comprises an evidence record that encompasses the sequence, molecular event, HLA restriction, presentation evidence with its coordinates on both axes of Table 1, the normal-tissue evidence base, cross-reactivity testing, immunogenicity including the phenotype of the induced CD4+ population, dominance behavior in the specified cassette, clonality relative to whole-genome doubling, population-specific eligibility, and clinical exposure. This comprehensive documentation facilitates a multidisciplinary review to elucidate the rationale behind the selection of each antigen and the reasons for the rejection of others.

The initial clinical portfolio should prioritize settings characterized by the highest signal-to-noise ratio: persistent viral oncoproteins in tumors driven by viruses, recurrent driver and frameshift neoantigens supported by direct presentation evidence, hematologic neoantigens detected through sensitive molecular techniques, and residual-disease states where recurrence is detectable within a practical timeframe. Targets should be developed further because the progression from malignant event to clinical endpoint can be substantiated, and predefined criteria exist to determine when to halt the program.

## 13. Limitations

This article presents five limitations that influence the degree of trustworthiness of its conclusions, with the first two being of the greatest significance.

The framework remains entirely unvalidated. The core-and-shell architecture has not yet been implemented within a clinical system, and its central operational premise, that early core priming during the manufacturing interval of a personalized shell enhances outcomes, has not been subjected to testing in a randomized comparison. Cassette-level immunodominance proposes a plausible alternative hypothesis: core-then-shell dosing may be actively detrimental relative to personalized vaccination alone. This hypothesis is articulated in Section 8 and Table 8 and remains unresolved based on current data; therefore, we present the architecture as a hypothesis.

The article contains no quantitative analysis, which is a deliberate reduction from an earlier version. We removed a prototype HLA eligibility submodel and its worked example because their inputs were constructed values, and the eligibility percentages and comparative gains they produced were properties of those inputs rather than findings about any population. Gate 3 is therefore specified but not computed, since the coverage calculation it requires needs measured allele and multilocus haplotype frequencies for named populations. For Gate 4, there is no model either: a composite persistence score was considered and is not offered, for the reasons in Section 6, and the variables the gate requires cannot at present be retrieved from the published record. The framework leaves two of its seven gates specified but unimplemented, which is a real limitation and is stated as one.

The synthesis is narrative rather than systematic. The author selected studies against the stated criteria rather than using a registered protocol with duplicate screening, and no quantitative synthesis was performed. The randomized evidence was not subjected to a formal Risk of Bias 2 assessment [10], and the selection and interpretation of evidence in Section 9 remain open to reasonable disagreement.

The evidence is preliminary. The melanoma outcome comes from a phase 2b trial involving 157 patients, using a one-sided alpha, with broad confidence intervals, immature overall survival data, and no confirmatory phase 3 report. The most compelling interception results stem from a single-arm study. The most insightful shared-library outcome reflects a design limitation rather than an accomplishment, and this asymmetry appears intentional.

The fifth limitation relates to applicability rather than validity. The framework necessitates evidence that is not routinely generated. Specifically, primary-tumor immunopeptidomics in the relevant HLA background, controlled demonstration of tumor cell destruction at endogenous antigen density, the cancer-cell fraction with a credible interval, presentation retention following a specified perturbation, and phenotyping of the induced CD4+ population are all achievable within specialized centers; however, none of these processes are part of standard practice. Notably, immunopeptidomics of primary tumors does not presently scale to routine use, representing the main practical obstacle to implementing the presentation-first discipline advocated herein. Section 6.1 clarifies the implications: for two traceable cases selected due to their favorable evidence base, the persistence variables could not be obtained in either instance. A framework that cannot be populated from existing literature is better regarded as a research agenda than a decision-making tool and is presented accordingly. A program adopting these criteria today would likely halt most candidates at Gate 2 because of a lack of evidence rather than evidence of absence, which aligns with current specifications and involves high practical costs. Accordingly, any group implementing this framework should determine in advance which gates will be sufficiently funded and formalize this decision, rather than allowing such choices to occur implicitly.

Finally, the author discloses a financial interest in the category of products described by this framework, as stated below. Readers are encouraged to evaluate the argument accordingly; the falsification conditions presented in Table 8 are partially provided for this purpose: they constitute the criteria on which the author’s own framework should be dismissed.

## 14. Conclusions

The future of cancer vaccination resides neither in a universal vaccine nor in unlimited personalization, but in precise immunization: the right antigen, in the right HLA context, at the right disease stage, on the appropriate platform, with the correct immune modulation, assessed against the suitable endpoint. Shared tumor-specific libraries aim to provide rapidity, consistency, and accessibility, none of which have yet been empirically demonstrated in clinical settings. Personalized neoantigens offer coverage when shared targets are inadequate. Collectively, they constitute a core-and-shell system that is biologically selective, operationally adaptable, and capable of being audited.

Messenger RNA accommodates this architecture through its modularity, multivalency, rapid manufacturing, and lack of any designated integration mechanism. The critical factor is not the complexity of the construct but whether the encoded peptide is sufficiently specific, properly presented, clonally diverse, persistent, dominant within its cassette, and immunologically effective to be meaningful. The Phase 2b recurrence-free survival signal observed in melanoma warrants cautious optimism. Conversely, the failure of immunodominance in a well-designed shared cassette necessitates prudence. A framework unable to encompass both results simultaneously does not adequately describe this field.

A mature discipline will supplant lists of attractive antigens with evidence that is carefully governed, replace fixed schedules with biological states that are measured accurately, substitute empirical combinations with diagnosed immune failures, and shift from population-average libraries to explicit distributional choices derived from measured data. The predictions presented here serve as invitations for testing rather than definitive claims to acceptance. Table 8 delineates the criteria that would refute each of these predictions, including the architecture itself. If these predictions withstand testing, they will transform cancer vaccination from a series of bespoke experiments into an adaptive therapeutic system. Conversely, if they fail, the manner of their failure will specify which assumption was incorrect, representing the extent of insight that a framework can offer prior to data collection.

## Figures and Tables

**Table 1:** This document uses the two-axis evidence classification system throughout. Axis 1 documents the study design category and is determined solely by the study’s design, independent of the measured outcomes. It does not function as a certainty rating; instead, assessing certainty also involves evaluating risk of bias, imprecision, indirectness, inconsistency, and selective reporting, the limitations of which are articulated in Section 9. Axis 2 records the outcome domain; it is not organized in a ranked hierarchy, and a single study may have multiple domain codes concurrently. The direction of the outcome, whether positive or negative, is documented separately, thereby facilitating the distinction between a negative randomized trial and a positive one, despite both sharing the same design code.

Axis 1: Study-Design Category (Design Only, Not a Certainty Rating)	Axis 2: Result Domain (Not a Hierarchy; Multiple Codes Permitted)
RCT. Randomized controlled trial	NP. Natural presentation. Immunopeptidomics establishes that the peptide–HLA complex exists on tumor
PNR. Prospective nonrandomized clinical study, whatever its primary endpoint	IR. Immune recognition. T-cell assays establish that the epitope is recognized whether on synthetic peptide or on engineered targets
REX. Retrospective or exploratory clinical analysis, including post hoc and secondary analyses	FR. Functional recognition. Killing of native, unmanipulated tumor at endogenous antigen density
PRE. Preclinical model or ex vivo human system	MR. Molecular response. ctDNA or disease-specific molecular clearance
CMP. Computational or in silico analysis	CA. Clinical activity. Objective response, or occurrence or regression of lesions
	CB. Clinical benefit. Survival, recurrence-free survival, or incidence
	PR. Prediction only

**Table 2:** Differentiation between validated evidence and the propositions of this article. The left column delineates findings substantiated by the cited primary literature. The right column outlines proposals associated with the current framework, none of which have been empirically tested.

Established by the Cited Evidence	Proposed Here, and Not Yet Tested
Prophylactic vaccination against hepatitis B and human papillomavirus reduces the incidence of the associated cancers [1,2,12]	That an analogous benefit follows for therapeutic vaccination against a nonviral tumor. Cause prevention is treated here as a boundary case outside the gate framework
Individualized mRNA neoantigen therapy with pembrolizumab prolonged recurrence-free survival in a randomized phase 2b trial in resected melanoma [13]	That a pre-manufactured shared library will do likewise. No shared library has shown clinical benefit in any setting
Epitopes encoded in one cassette compete: a 20-antigen shared cassette produced responses directed at encoded TP53 epitopes rather than at the KRAS neoantigens the tumors carried [14]	That cassette composition can be optimized prospectively using the gate sequence and the persistence criteria proposed here
A 209-peptide off-the-shelf frameshift cassette was safe and immunogenic in all 37 evaluable Lynch syndrome carriers [15]	That interception is the setting in which a shared library will first show clinical benefit
Neoantigen-specific Tr1 CD4+ cells can suppress antitumor immunity [16], and some neoantigens drive tumor growth rather than control it [17]	That routine phenotyping of the induced CD4+ population improves candidate selection
Individualized vaccines can be manufactured and delivered, with reported production intervals from several weeks to a median of about twenty weeks [18,19]	That a pre-manufactured core given during that interval improves outcome, rather than imprinting a hierarchy that degrades the shell

**Table 3:** The four distinct objectives of cancer vaccines, along with the evidentiary standards required for each. Cause prevention is a boundary case and is excluded from the seven-gate framework described in Section 5, as it occurs before tumor development and does not depend on peptide presentation by malignant cells.

Objective	Target State	Acceptable Risk	Preferred Endpoint	Dominant Failure Mode	Best Current Logic
Cause prevention	Before oncogenic infection	Very low	Cancer incidence	Few, where a stable foreign antigen exists	Block a stable upstream cause before immune escape can evolve
Interception	Premalignancy or high-risk carrier state	Low	Advanced lesion or cancer incidence	Cross-reactivity in a person without cancer	Target recurrent early tumor-specific events such as frameshift peptides
Residual disease	After definitive therapy	Moderate	Molecular clearance; recurrence-free survival	Subclonal antigen escape	Address small, evolving clone populations while the immune context is least suppressed
Established cancer	Measurable invasive disease	Higher	Objective response; duration; survival	Mature suppressive ecosystem; HLA loss	Vaccination plus correction of the dominant, diagnosed immune failure

**Table 4:** Antigen classes, escape properties, and the minimum evidence required before clinical use.

Antigen Class	Example	Shared Across Patients	Fitness Cost of Loss	Minimum Evidence Required
Viral oncoprotein	HPV16 E6/E7; HBV; Merkel cell polyomavirus T antigen	High	High, where expression is obligate	Confirmed viral driver status; presentation on patient HLA; construct rendered non-transforming
Recurrent driver neoepitope	*KRAS* G12D/G12V; IDH1 R132H; histone H3 K27M	Moderate to high	High, where the driver is truncal	Direct peptide–HLA detection; clonality; T-cell recognition that discriminates mutant from wild type
Shared frameshift peptide	Microsatellite-unstable indels (for example *TGFBR2*); *NPM1*; *CALR*	High within a molecular subgroup	Moderate	Recurrence across lesions; immunopeptidome or intact-tumor recognition; normal-tissue exclusion within a declared evidence base
Fusion junction	DNAJB1-PRKACA; EWS-FLI1; BCR-ABL	Low to moderate	High, where the fusion is the driver	Junction-spanning peptide detected on HLA, not inferred from the fusion transcript
Private neoantigen	Patient-unique missense or indel	None	Variable; often low if subclonal	Clonality; expression at RNA level; orthogonal T-cell assay with wild-type control
Tumor-associated self-antigen	MAGE-A3; MUC1; PSA; telomerase	High	Low	Separate track. Tolerance and on-target off-tumor toxicity must be addressed explicitly

**Table 5:** The antigen-to-benefit gates. Failure at any gate warrants stopping evaluation of a candidate or reclassifying it. The final column presents the recommendations outlined in this article. Regulatory authorities or consensus standards do not mandate these; interpret them as recommendations. These gates apply only to antigen-defined, HLA-restricted vaccines.

Gate	Question	Minimum Evidence	Recommended Response to Failure
1. Specificity	Does the peptide–HLA target have any detected presentation on essential normal tissue?	Normal-tissue multi-omics within a declared evidence base; proteome-wide similarity search; cross-reactivity testing in primary human cells	Recommend stopping, or reclassifying as tumor-associated under a separate safety standard
2. Presentation	Is it naturally displayed by malignant cells?	Immunopeptidomics from primary tumor, or recognition of native tumor cells by antigen-specific T cells	Recommend against advancing on prediction alone
3. Coverage	How many tumors and how many people can present it?	Event prevalence, tumor expression, presentation probability, and HLA eligibility, computed jointly and reported for named populations from measured allele and haplotype frequencies. No coverage model is proposed here	Recommend defining a narrower subgroup, or adding modules whose restrictions extend eligibility in the least-served population
4. Persistence	Will the target survive immune pressure?	Clonality relative to whole-genome doubling; conservation across metastases; functional dependence; stability under interferon and therapy. Each is measured and reported in the units specified in Section 6. No ranking score is proposed	Recommend pairing with an independent persistent target whose escape route is defined and nonoverlapping
5. Immunogenicity	Can functional T cells recognize it selectively, and does it suppress anything?	Orthogonal T-cell assays with wild-type and irrelevant peptide controls; killing of native tumor; assessment of suppressive and regulatory outcomes	Recommend redesign or rejection; absence of a suppressive response should be shown rather than assumed
6. Product	Can it be made reproducibly with linked potency, and how does it behave in its cassette?	Validated process; mechanism-linked potency assay; cassette-level immunodominance testing	Recommend optimizing the construct, the cassette composition, or the platform
7. Benefit	Does it improve the correct clinical outcome?	Randomized controlled trial in the intended disease state	Recommend against substituting an immune response for efficacy

**Table 6:** Measurement specifications for the gates that precede and constitute the persistence assessment. Gate 1 pertains to specificity, and Gate 2 pertains to presentation; both are qualitative pass-or-fail checkpoints, and neither is scored. The four persistence variables are mandatory: a candidate for which any of C, M, D, or R has not been measured is not characterized in terms of persistence, is not compared with other candidates that are, and receives no imputed value, as defaulting to missing evidence is not permitted. Each value must be documented with an uncertainty interval, along with the assay and, where applicable, the perturbation that produced it. This specification does not specify numeric bins, weights, or thresholds. A program using this specification must establish its thresholds prospectively, justify them in the context of the disease, and record them before evaluating candidates.

Criterion	Role	Definition and Required Reporting	If the Evidence is Absent
Presentation evidence	Gate 2 (pass or fail)	Admissible: (i) immunopeptidomic detection on primary tumor in the relevant HLA background; or (ii) killing of native, unmanipulated tumor at endogenous antigen density WITH target specificity and HLA restriction demonstrated, by target knockout, HLA knockout or blockade, mutant-versus-wild-type controls, and rescue. Killing alone is not admissible. Not admissible: detection only in an engineered, transduced, or monoallelic overexpression system; immunogenicity against synthetic peptide only; prediction only. Admissibility is Boolean	Not admissible. Do not advance
Tumor specificity	Gate 1 (pass or fail)	Admissible only where a proteome-wide similarity search has been completed AND a primary-human-cell cross-reactivity assay is negative. Similarity hits confined to non-essential tissue are recorded and carried forward as a stated residual risk. Any hit in essential tissue, and any positive primary-cell reactivity, fails the gate. No numeric similarity scale is proposed, because the consequence of a hit is determined by the tissue in which it falls	Not admissible. Do not advance. Absence of a search is not evidence of absence
Malignant-cell prevalence (C)	Persistence variable (mandatory)	The cancer-cell fraction: the purity- and ploidy-adjusted proportion of malignant cells carrying the event, recorded as a point estimate with a credible interval. This is deliberately NOT called clonality. Where a program imposes a clonality requirement, it is applied to the LOWER credible bound, at a threshold set by disease context and stated in advance	Not characterized. Do not compare. Do not impute
Metastatic conservation (M)	Persistence variable (mandatory)	Fraction of sampled metastatic or multiregion sites retaining the event, reported together with the number of independent sites sampled. A value derived from fewer than three independent sites is reported as such and is not comparable with a value derived from three or more	Not characterized. Do not compare. Do not impute
Functional dependence (D)	Persistence variable (mandatory)	The degree to which the transformed phenotype requires expression of the encoded product, established by a named external dependency measurement with the source and any normalization stated. Driver status inferred from recurrence alone does not establish dependence. Obligate viral oncoproteins, truncal drivers supported by functional-genomic dependency data, context-dependent drivers, lineage antigens, and passengers are distinguished by the evidence supporting them, not by a numeric bin	Not characterized. Do not compare. Do not impute
Presentation retention (R)	Persistence variable (mandatory)	Proportion of baseline presentation retained after a named perturbation, such as interferon exposure or the intended therapy. The perturbation and the assay must be stated. Peptide–HLA complex stability and antigen-processing retention are distinct mechanisms and are reported separately where both are measured	Not characterized. Do not compare. Do not impute

**Table 7:** Retrieval of the persistence variables specified in Table 6 from the published record, for two traceable antigen–cancer–HLA cases selected for their favorable evidence base. A variable is marked as retrievable only when a published measurement in the units required by the specification was identified. No values were imputed. Neither case can be characterized for persistence. This tests whether the specification can currently be populated from the literature, not whether any ranking method is effective.

Variable	Case 1: *RPL18* p.Met53Ile, Primary Cervical Adenocarcinoma	Case 2: *KRAS* G12D on HLA-C*08:02, Pancreatic Ductal Adenocarcinoma	Status
Gate 1, tumor specificity	Tumor specificity established by subtraction against a personalized healthy proteome; no proteome-wide similarity search or primary-human-cell assay reported	Mutant versus wild-type discrimination reported; no primary-human-cell cross-reactivity assay in the form the specification requires	Not established to the standard the specification requires, in either case
Gate 2, presentation	Detected by mass spectrometry in the primary-tumor immunopeptidome in the relevant HLA background	Peptide–HLA presentation reported; primary-tumor immunopeptidomic detection not consistently reported across studies	Admissible in case 1; arguable in case 2
C, malignant-cell prevalence	Not reported as a purity- and ploidy-adjusted cancer-cell fraction with a credible interval	Truncal in pancreatic ductal adenocarcinoma, but not reported in the required units with an interval	Not retrievable
M, metastatic conservation	Single lesion analyzed; fewer than the three independent sites the specification requires	Not reported for the peptide–HLA complex across three or more independent sites	Not retrievable
D, functional dependence	Passenger on the available evidence	Truncal driver on the available evidence	Describable qualitatively; the named dependency measurement and normalization the specification requires are not supplied
R, presentation retention	Not reported after any named perturbation	Not reported after any named perturbation	Not retrievable
Persistence assessment	Cannot be characterized	Cannot be characterized	0 of 2 cases characterizable

**Table 8:** The four predictions, their primary observable, the definitive experiment, and the result that would invalidate each. The final row indicates the competing hypothesis that would disprove the architecture itself.

Prediction	Primary Observable	Decisive Experiment	Falsified If
1. Presentation-first selection outperforms prediction-first selection	Native-tumor recognition per encoded target at endogenous antigen density	Blinded comparison of equal-sized panels in matched HLA backgrounds	Prediction-first panels achieve equivalent native-tumor recognition
2. Measured antigen persistence predicts durable control better than immunogenicity alone	Durable molecular clearance	Prospective validation with measured, not assigned, component inputs against ctDNA, serial biopsy, and recurrence	Response magnitude predicts durable control as well as the measured persistence variables
3. Evolutionary trapping requires escape-route independence	Time to clonal outgrowth as a function of measured independence	Barcoded heterogeneous organoid and immune-competent models with each dependency layer perturbed individually	Redundant pairs delay escape equally
4. State-based dosing and diagnosed-failure combinations outperform fixed regimens	Durable control per unit cumulative toxicity	Randomized biomarker-directed versus fixed-schedule trial with decision rules prespecified in protocol	Adaptive assignment yields no improvement in the control-to-toxicity ratio
Competing hypothesis that would refute the core-and-shell architecture (not one of the four numbered predictions)	Clonal breadth and dominance hierarchy across every encoded antigen after sequential dosing	Four-arm trial: shell alone; simultaneous core and shell; core then shell; shell then core	Core priming imprints a hierarchy that diminishes shell responses, in which case core-then-shell sequencing is inferior to shell alone and the architecture fails

## Data Availability

No dataset was generated or analyzed for this article. The two traceable cases examined in Section 6.1 and all clinical data discussed in the manuscript are drawn from the cited publications.
